# Statistical Modeling for Quality Assurance of Human Papillomavirus DNA Batch Testing

**DOI:** 10.1097/LGT.0000000000000391

**Published:** 2018-05-04

**Authors:** Emily N. Beylerian, Rose C. Slavkovsky, Francesca M. Holme, Jose A. Jeronimo

**Affiliations:** 1 PATH, Seattle, WA; and; 2 Global Coalition Against Cervical Cancer, Arlington, VA

**Keywords:** HPV DNA testing, cervical cancer, screening, quality assurance, Latin America, low-resource settings

## Abstract

**Objectives:**

Our objective was to simulate the distribution of human papillomavirus (HPV) DNA test results from a 96-well microplate assay to identify results that may be consistent with well-to-well contamination, enabling programs to apply specific quality assurance parameters.

**Materials and Methods:**

For this modeling study, we designed an algorithm that generated the analysis population of 900,000 to simulate the results of 10,000 microplate assays, assuming discrete HPV prevalences of 12%, 13%, 14%, 15%, and 16%. Using binomial draws, the algorithm created a vector of results for each prevalence and reassembled them into 96-well matrices for results distribution analysis of the number of positive cells and number and size of cell clusters (≥2 positive cells horizontally or vertically adjacent) per matrix.

**Results:**

For simulation conditions of 12% and 16% HPV prevalence, 95% of the matrices displayed the following characteristics: 5 to 17 and 8 to 22 total positive cells, 0 to 4 and 0 to 5 positive cell clusters, and largest cluster sizes of up to 5 and up to 6 positive cells, respectively.

**Conclusions:**

Our results suggest that screening programs in regions with an oncogenic HPV prevalence of 12% to 16% can expect 5 to 22 positive results per microplate in approximately 95% of assays and 0 to 5 positive results clusters with no cluster larger than 6 positive results. Results consistently outside of these ranges deviate from what is statistically expected and could be the result of well-to-well contamination. Our results provide guidance that laboratories can use to identify microplates suspicious for well-to-well contamination, enabling improved quality assurance.

## Abstract 2

Supplemental digital content is available in the text.

Cervical cancer is caused by infection by oncogenic human papillomaviruses (HPVs) and is a leading cancer killer of women in low-resource settings. This neoplasia is preceded by persistent infection with HPV, which can lead to precancerous lesions several years before development of invasive cancer. Detection of oncogenic HPV genotypes in cervical or vaginal samples allows screening programs to identify women at higher risk for developing this disease.^1–3^ Human papillomavirus DNA testing has a higher sensitivity than other screening methods and allows longer screening intervals.^4^ Recently, a low-cost HPV DNA test (*care*HPV, QIAGEN, Gaithersburg, MD) was introduced through the public health systems in Guatemala, El Salvador, Honduras, and Nicaragua with assistance from PATH, a nongovernmental organization, and in collaboration with local partner organizations.^5^ Despite the accuracy of HPV DNA screening, we observed unexpected test results from some of the Central American laboratories, including higher-than-predicted numbers of positive results.

The assay is carried out in a 96-well microplate by a validated technician. The testing procedure includes manual steps such as pipetting specimens and reagents into microplate wells; washing, decanting, and blotting the microplate; and transferring the microplate from the bench top to a heater/shaker and luminometer. Some manual steps have been identified by the test manufacturer as potential opportunities for well-to-well contamination,^6^ whereby material from a positive sample in one well is transferred to an adjacent well and produces a false-positive result in that well. Although the risk of contamination is lower for a signal amplification test (such as *care*HPV) than a polymerase chain reaction test, the manual procedures such as decanting or washing the plate could result in a significant amount of material being moved between wells.

The concerning observations from Central America were some microplates with a higher positivity rate than expected based on the predicted oncogenic HPV prevalence for the geographic region and multiple and/or large clusters of positive test results in the microplate. We hypothesized that statistical modeling of the distribution of positive results under expected positivity rates for the population would demonstrate whether these observations were consistent with well-to-well contamination. This would enable us to create parameters that could be useful in identifying microplates suspicious for contamination.

The objective of this modeling study was to simulate microplate assay results based on discrete oncogenic HPV prevalence to investigate 3 categories for quality assurance (quality categories): (1) number of positive wells per microplate, (2) number of clusters of positive wells per microplate, and (3) size of the largest cluster per microplate and to compare statistics from the simulation results with observed test results for each category.

## MATERIALS AND METHODS

We started by carrying out a literature review of articles published between 1996 and 2016 pertaining to women in Latin America and the Caribbean to determine the oncogenic HPV prevalence in Central America for use in our simulation algorithm. Search terms included (“HPV” OR “human papillomavirus” OR “papillomavirus infection”) + (“cervical cancer” OR “uterine cervical neoplasms”) + (“prevalence” OR “epidemiology”). Of 216 articles retrieved, we selected studies that (1) screened a general population using validated HPV testing, (2) provided the age of women screened, and (3) had a study size of more than 400 women. The articles we used reported prevalence using tests that detected 13 to 18 oncogenic HPV genotypes. When possible, we used the reported prevalence for women closest to the target age range of 30 to 64 years used by the Central American screening programs. The prevalence of oncogenic HPV infection from this review ranged from 12% to 16%. This range was used for our modeling exercise.^7–13^

Statistical analysis for this project was carried out in the programming language R. We wrote an algorithm to simulate test results stochastically, assuming discrete HPV prevalence of 12%, 13%, 14%, 15%, and 16%—where each prevalence is the simulation condition—and to execute the following analytical steps for each (see Appendix A for more details on the algorithm and examples of the R code).

The algorithm first created the analysis population of 900,000, representing women of the target age for screening. We specified 900,000 draws from the binomial distribution (positive or negative for HPV), which assumes that each draw has a consistent and independent probability of being positive. The probability of each draw equaled the simulation condition (discrete HPV prevalence). We selected 900,000 draws to simulate the individual results of 10,000 microplate assays. This generated a vector of results where each entry was either 1 or 0 (a positive or negative test result, respectively).

**FIGURE 1:**
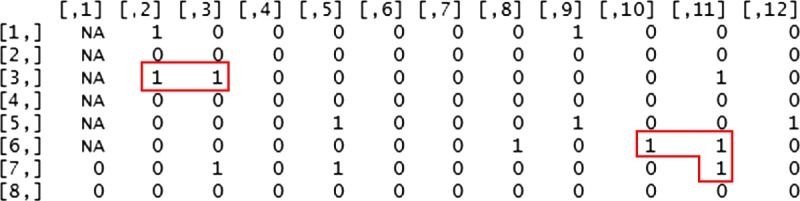
Output for one 12 × 8 matrix representing a hypothetical assay microplate. Clusters of positive results are outlined in red. See Appendix A for details of the algorithm.

Because each 96-well microplate contains 6 control wells (3 positive, 3 negative) and 90 test wells, the vector was divided into 10,000 groups each containing 90 simulated results, and each group had 6 “not applicable” (NA) values appended to the beginning of the vector to represent the control wells. The 96-element vectors were then reshaped to become 12 × 8 matrices to represent 96-well assay microplates in shape and structure (see Figure 1). The process by which each matrix was shaped followed the order in which laboratory technicians fill the microplate with calibrators and specimens, beginning in the top-left corner and moving columnwise from left to right. We used these matrices as a basis for calculating aggregate measures to be compared with real microplate test results (see Figure 1).

After assembling the 10,000 96-cell matrices, the algorithm aggregated the results to create analytical measures in each quality category.

As noted previously, the quality categories planned for analysis were (1) number of positive cells per matrix, (2) number of clusters of positive cells per matrix, and (3) size of the largest cluster per matrix, where the matrices represent 96-well microplates. Thus, the first tabulation was the number of positive cells per matrix for all matrices in the simulation, giving a distribution of expected results under the simulation conditions (when viewed as a percentage of cells that are positive, this is roughly equal to the HPV prevalence defined as the simulation condition).

Next, we defined a cluster as 2 or more positive cells horizontally or vertically adjacent and defined the size of a cluster to be the number of cells that make up the cluster (see Figure 1). Based on our observation of the manual assay steps, including pipetting reagents and manipulating the microplate, and considering the shape of the microplate, we determined that it was improbable for material to be carried from one well to a diagonal well without also transferring material to an adjacent well; therefore, we did not designate diagonal cells as a site for possible contamination. To generate data for the analysis of these quality categories, the algorithm identified the clusters in each matrix of simulated results to enable their tabulation and analysis. This was carried out by first identifying pairs of adjacent positive cells and then grouping those pairs into clusters.

To identify pairs of adjacent positive cells, the algorithm assigned an identification (ID) number between 1 and 96 to each matrix cell, including those cells with an NA value. The ID numbers were assigned consecutively in the same order by which the matrices were populated, beginning in the top-left corner and moving columnwise from left to right. The algorithm then located pairs of adjacent positive cells by calculating the difference between cell ID numbers. When the difference between the cell ID numbers was equal to ±8, the cells were horizontally adjacent. When the difference between the cell ID numbers was equal to ±1, the cells were vertically adjacent. To ensure that perimeter cells with a difference of ±1 that were located in different columns were not counted as adjacent, the algorithm removed these 11 pairings from consideration as adjacent positive results. The algorithm then returned a list of pairs of adjacent positive cells.

Next, the algorithm considered every combination of pairs in the matrix and combined those with shared cell IDs to identify complete clusters in each matrix. The algorithm then counted the number of clusters per matrix and the size of the largest cluster per matrix to give a distribution for the expectation of these metrics under the simulation conditions.

The algorithm generated results for each matrix in the simulation and created curves of normal distributions for each parameter evaluated (number of positive cells, number and size of clusters in the matrices); the simulation results yielded the frequency distribution in each quality category for the simulation conditions of 12%, 13%, 14%, 15%, and 16%. In the Discussion, we convert these parameters to numbers and clusters of wells in microplates and convert the conditions (12%–16%) to oncogenic HPV prevalence.

To confirm the sample size, we inspected the distribution of results in each quality category when using sizes ranging from 1 matrix to 10,000 matrices. All chosen measures across simulation conditions stabilize well before reaching 10,000 matrices, giving us confidence in the simulation sizes.

In addition, to provide guidance for screening programs in low-resource settings outside of Central America, we ran the analysis described in this article for prevalence rates 8% to 11% and 17% to 23% as a supplemental analysis.^14^

### Role of the Funding Source

The funding source had no involvement in the development of the methodology.

## RESULTS

We present our results for the 3 parameters that were evaluated as tables with numerical values for each condition and parameter.

### Number of Positive Cells Per Matrix

**FIGURE 2:**
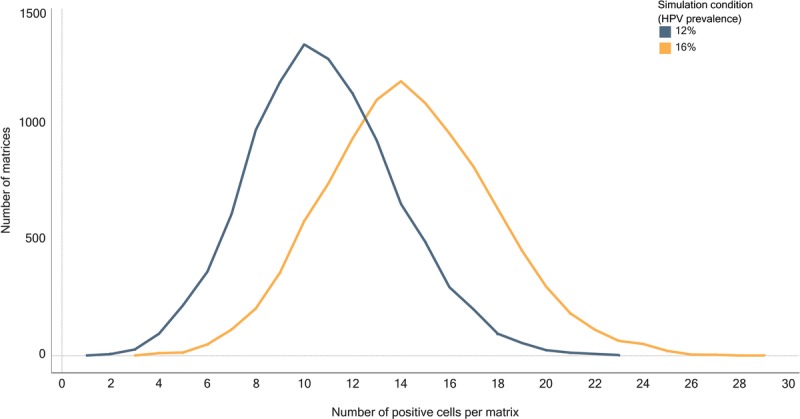
Frequency distributions of the number of positive cells under simulation conditions of 12% and 16% (simulated HPV prevalence). Each simulation results in 10,000 matrices. As the simulated prevalence increases, the distribution range shifts to the right.

For a simulation condition of 12% (i.e., simulating HPV prevalence of 12%), the number of positive cells per matrix ranged from 1 to 23, with 95% of the matrices having between 5 and 17 positive cells (see Table 1). For a simulation condition of 16%, the number of positive cells per matrix ranged from 3 to 29, with 95% of the matrices having between 8 and 22 positive cells, and for conditions between 12% and 16%, results were intermediate. As the simulation condition increased, the middle 95% of the data points shifted toward higher values (see Table 1), and the distribution curve shifted to the right, as did the mode for positive cells found in the distribution (see Figure 2).

**TABLE 1:**
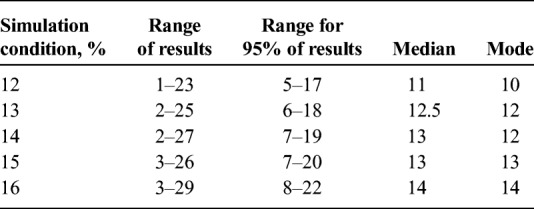
Results for Number of Positive Cells Per Matrix by Simulation Condition (12%–16%)

### Number of Cell Clusters Per Matrix

For a simulation condition of 12%, the number of cell clusters per matrix ranged from 0 to 7, with 95% of the matrices in the simulation having between 0 and 4 clusters. For a simulation condition of 16%, the number of cell clusters per matrix ranged from 0 to 8, with 95% of the matrices having between 0 and 5 clusters (see Table 2).

**TABLE 2:**
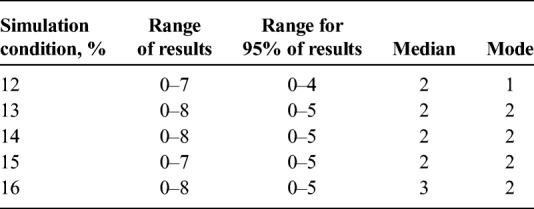
Results for Number of Clusters Per Matrix by Simulation Condition (12%–16%)

### Size of the Largest Cell Cluster Per Matrix

For a simulation condition of 12%, the size of the largest cell cluster per matrix ranged from 1 (no cluster) to 9 positive cells, with 95% of the matrices in the simulation having a largest cluster size between 1 and 5 positive cells. For a simulation condition of 16%, the size of the largest cell cluster per matrix ranged from 1 (no cluster) to 11 positive cells, with 95% of the matrices in the simulation having a largest cluster size between 1 and 6 positive cells (see Table 3). As the condition of the simulation increases, the frequency distribution of results shifts toward larger numbers for all quality categories.

**TABLE 3:**
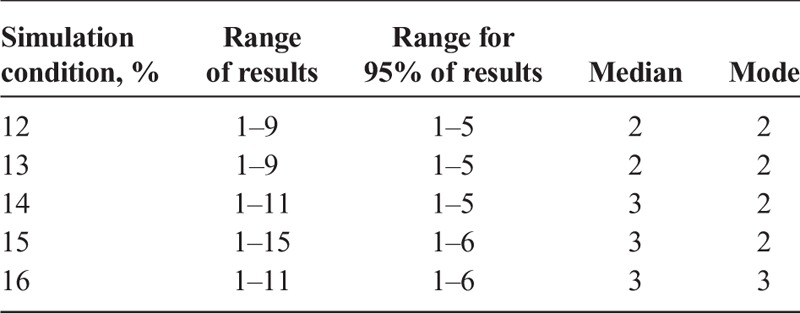
Results for the Size of the Largest Cluster Per Matrix by Simulation Condition (12%–16%)

### Results for Additional Prevalence Ranges

For simulation conditions 8% to 11% and 17% to 23%, the frequency distribution of results shifts toward lower numbers for all quality categories as the condition of the simulation decreases and higher numbers as the condition increases (Supplemental Tables 1 http://links.lww.com/LGT/A90, 2 http://links.lww.com/LGT/A91, and 3 http://links.lww.com/LGT/A92).

## DISCUSSION

To discuss our results in terms of a laboratory setting, instead of using the modeling terminology of *cells* and *matrices*, we now will refer to *results* or *wells* and *microplates*. To the best of our knowledge, this is the first statistical modeling exercise conducted for evaluating the likelihood of well-to-well contamination in 96-well microplates. Our interpretation of the modeling results as applied to laboratory practice is that microplate results that are not consistent with the simulations are either consistent with well-to-well contamination or do not hold with the assumptions of the simulation.

Our results suggest that HPV DNA screening programs in regions with an oncogenic HPV prevalence of 12% to 16% can reasonably expect to observe between 9 and 17 positive results per microplate in approximately 50% of assays and between 5 and 22 positive results per microplate in approximately 95% of assays. These ranges represent the exterior limits of the middle 50% and middle 95% of the data points when comparing across the 12% to 16% prevalence simulations without aggregating. We would expect the most frequent number of positive results per plate to be between 10 and 14. Results consistently outside the range of 5 to 22 positive results deviate from what is statistically expected and could be the result of well-to-well contamination. Similarly, our findings suggest that these same screening programs should anticipate between 0 and 5 positive clusters per microplate, representing at least 95% of all data points of the simulation results under each prevalence condition. Our results also indicate that in general, clusters are likely to be present. Furthermore, screening programs in Central America would not anticipate a cluster larger than 6 positive wells per microplate, with most clusters having 2 to 3 positive wells. Cluster sizes that are not within the range of the modeled results would be suspicious for well-to-well contamination. Screening programs in other regions may anticipate lower or higher numbers of positive wells and clusters, as well as smaller or larger sizes of the largest cluster per microplate, depending on local HPV prevalence.

These modeled results can be used to provide quality assurance parameters for screening programs implementing HPV tests conducted in batches on a 12 × 8 microplate with 6 control wells when microplates are run with 90 samples. For example, in Central America, screening programs may decide that microplates with greater than 22 positive results, with more than 5 clusters, or containing clusters of more than 6 positive wells are suspicious of well-to-well contamination and do not meet quality standards.

**FIGURE 3:**
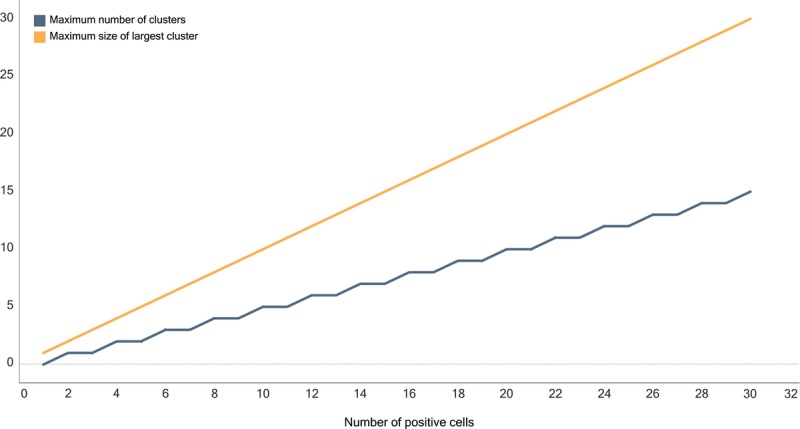
Maximum possibilities for the number of clusters and the size of the largest cluster when the number of positive cells in a matrix is constrained.

While presented individually, the 3 quality categories are interdependent, and therefore, constraining the allowable outcomes in one category also constrains the outcomes in the other 2. For example, when constraining the total number of positive cells, the number of possible clusters is constrained at half the number of positive cells and the possible size of the largest cluster is constrained at the total number of positive cells (see Figure 3). Despite this interdependency, it is necessary to consider all 3 categories in the context of contamination because constraining one category leaves enough freedom in the remaining categories to allow for conditions suspicious of contamination.

Our analysis requires a known underlying oncogenic HPV prevalence, which is a constraint in areas where HPV screening is novel and prevalence is not yet established. It also is limited by the assumption that prevalence is constant throughout a community and, therefore, that samples from women screened together are not more or less likely to have the same results. However, studies have reported that prevalence is not always consistent throughout a community and can vary depending on factors such as age, geographic location, and HIV prevalence. In Central and South America, HPV prevalence is highest in women younger than 25 years, after which it decreases, and is lowest in women aged 35 to 44 years, before reaching a second peak in women older than 45 years.^15^ Prevalence may vary between rural and urban areas, and it correlates positively with HIV prevalence in a given population.^16–20^ Therefore, screening programs that develop quality assurance tools based on our analysis should reconsider periodically the characteristics of the population being screened and how this may impact expected HPV positivity rates in their assays.

This analysis is limited to providing statistical considerations for the likelihood of well-to-well contamination and does not diagnose causes of observed patterns and outlying positivity rates. For example, our results are not able to identify at which assay step well-to-well contamination may have occurred or, in a plate with a positivity rate below the lower normal limit, what factors may have contributed to these outlier results. Similarly, it does not account for other possible errors outside the test process such as tainted or mislabeled specimens, reagents, or calibrators. Furthermore, because of the nature of our defined quality categories, contamination in a plate that has a low but normal number of positive wells can go unnoticed if, for example, a sole cluster of positive wells does not meet the defined threshold for suspicion. For the quality category of number of positive wells per plate, we consider both upper and lower limits in this analysis. While an increased number of positive wells could be the result of well-to-well contamination, results that fall below the lower limit could indicate (1) a problem with the samples collected or how they were stored or (2) failure to pipette the accurate volume of sample into the assay microplate.

## CONCLUSIONS

Our analysis provides guidance that laboratories can use to identify microplates suspicious for well-to-well contamination. Each country or screening program will need to determine the range of acceptable results for each quality category (number of positive wells, number of clusters, and size of largest cluster) based on the oncogenic HPV prevalence in the community screened. Programs will also need to determine the action to be taken whether a microplate fails to meet quality standards, such as repeating the microplate assay or retraining technicians.

Quality assurance parameters developed for screening programs based on these results could be used to identify laboratory technicians who are in need of additional training. These parameters also can be used to develop a simple tool that laboratory technicians can reference when interpreting the results of each microplate, enabling them to monitor their own performance.

For Central America, we developed and piloted such a tool for laboratory technicians. Although results for most of the microplates observed in these screening programs have been within the middle 95% of data points, in cases where results have fallen outside this range, this tool has enabled local laboratory technicians to identify those microplates as suspicious for well-to-well contamination and has helped programs identify laboratory technicians in need of skills reinforcement. Further evaluation of the use of this tool in the field could provide additional guidance for cervical cancer screening programs in other countries.

As discussed as part of the limitations of this analysis, it may take time for screening programs initiating HPV testing to determine the expected oncogenic HPV prevalence in the region they are serving; therefore, programs should carry out periodic reassessments of the quality assurance parameters.

As countries with emerging economies adopt HPV DNA testing as a primary screening test for cervical cancer prevention, quality assurance programs are needed to ensure valid testing and the accuracy of results delivered to patients. A results interpretation tool such as the one discussed in this article is one example of how screening programs can use statistical analyses to establish parameters that can be adopted and implemented at the laboratory level, saving time and resources at the central program level.

## Supplementary Material

SUPPLEMENTARY MATERIAL

## APPENDIX A

All analyses for this project were carried out in the statistical programming language R, Version 3.2.4, with which we wrote an algorithm to conduct the simulations and characterize results. The simulation algorithm is as follows. The binomial distribution *B*(*n,p*) describes the probability of observing some number of successes in a group of *n* observations, where each successful outcome has the same independent probability *p*. Simulated test results were drawn as random variates from the binomial distribution. Each simulation generated 10,000 matrices of test results, representing the results from 10,000 microplates. Multiple simulations were run with different independent probabilities (the simulation condition), representing communities with different underlying HPV prevalence between 12% and 16%.

The backbone of simulating from the binomial distribution in R is the *rbinom* function from the *stats* package; we specified one trial of 900,000 observations, where each observation has a consistent probability *p* of being positive. This generated a vector where each component is either 1 or 0 (a positive or negative test result, respectively).

Using the *split*, *lapply*, and *matrix* base R commands (see code snippet below), the single vector was modified to resemble 10,000 of the 96-well microplates described above. The vector was reshaped into a list with 10,000 elements, where each list element contained 90 of the simulated results. Each list element had 6 NA values appended to the beginning of the vector of 90 results and was reshaped from a vector to a 12 × 8 matrix (filled columnwise). The resulting matrices represent assay microplates in shape and structure.

cases < − rbinom (*n* = 900,000, size = 1, prob = p)

caselist < − split(cases, f = ceiling(seq_along(cases)/90))

plates < − lapply(X = caselist, FUN = function(*x*){matrix(data = c(rep(NA, 6), *x*), nrow = 8, ncol = 12, byrow = FALSE)})

R code snippet for simulating 10,000 assay plates of test results.

To achieve the first analysis goal of characterizing the number of positive cells per matrix, the algorithm took the sum of all the cells in a matrix.

The second analysis goal was to characterize the number of clusters of positive cells per matrix. First, the algorithm located pairs of positive cells in the matrices by assigning a cell ID number to each matrix cell, identifying the position of each positive cell, and identifying each possible combination of positive cell IDs in each matrix. Because the cell ID numbers were assigned columnwise from the top left, if the difference between any pair of positive IDs from a matrix was equal to 1, the 2 positives were vertically adjacent in a matrix, and if the difference was 8, the 2 positives were horizontally adjacent in a matrix. To ensure that consecutive positives at the top and bottom borders of the plate were not counted as adjacent, any combinations of bottom- and top-border cells were removed from consideration as adjacent positives. Subsetting the positive ID combinations to only those whose difference was 1 or 8 returned a list of adjacent positives.

A group of 3 positive cells in a row would initially be identified as 2 different adjacent pairs. Therefore, after compiling an R list of adjacent positive cell pairs for each matrix in the simulation, the adjacent pairs were grouped into clusters. Positive cell IDs were defined to be in the same cluster if there existed a path between them only along adjacent positive cells.

Any 2 adjacent pair vectors with a positive cell ID in common must be touching and therefore part of a single cluster. Starting with the positive cell ID numbers in the first vector in the list (representing 1 adjacent pair in the matrix), the following vectors in the list were checked for cell IDs in common with the first pair. Cell IDs from the subsequent pair were added to the IDs in the first vector, creating a cluster (and repeating 1 of the ID numbers). After compiling all the cell clusters, duplicate ID numbers were removed. As a result, each element in the list represented a single cluster of positive cells in the matrix; within each list element was a vector containing the matrix position IDs involved in the cluster.

clusterlist < − lapply(diffpairs, FUN = function(*y*){betwnpos < − apply(*y*, 2, diff)

adjpairs < − *y*[, which(betwnpos == 1 | betwnpos == 8)]

clusterlist < − as.list(as.data.frame(adjpairs))

# combine all adjacent pairs that share positive IDs

npairs < − length(clusterlist)

dupind < − *c*()

if(npairs > 1){

for(*i* in 1:(npairs − 1)){

for(*j* in (*i* + 1):npairs){

if(any(clusterlist[[*i*]] %in% clusterlist[[*j*]])){

# add cluster lists that share wells

clusterlist[[*i*]] < − c(clusterlist[[*i*]], clusterlist[[*j*]])

dupind < − *c*(dupind, *j*) # keep track of list elts that get duplicated

}

}

}

if(length(dupind) > 0){

clusterlist < − clusterlist[−dupind] # remove repeated clusters

clusterlist < − lapply(clusterlist, unique) # remove repeated wells

}

}

clusterlist

})

The third analysis goal was to characterize the expected size of positive cell clusters (the numbers of cells that make up the clusters). The algorithm measured the size of clusters by taking the length of each element in the list of clusters on a matrix. We recorded the size of each cluster and noted the size of the largest cell cluster in each matrix.
