# Supplementary material for: Statistical Modeling for Quality Assurance of Human Papillomavirus DNA Batch Testing
Source: J Low Genit Tract Dis. 2018 May 4;22(3):219–24. doi: 10.1097/LGT.0000000000000391 (PMC6023602; doi:10.1097/LGT.0000000000000391)
Supplement: SUPPLEMENTARY MATERIAL [file lgt-22-219-s001.docx]

Appendix 1. Guideline IId. <24 years old (CIN 2,3)

PRACTICE POINTS:

- Guidelines for the management of young women from 21-24: Given a compliant patient and a reliable follow up system, it is reasonable to follow young women for up to 24 months or up to the age of 24 years (whichever comes first). Treatment recommendations are solely the responsibility of the treating physician. Follow up colposcopy exams should include biopsy and ECC.
