# Supplementary material for: Statistical Modeling for Quality Assurance of Human Papillomavirus DNA Batch Testing
Source: J Low Genit Tract Dis. 2018 May 4;22(3):219–24. doi: 10.1097/LGT.0000000000000391 (PMC6023602; doi:10.1097/LGT.0000000000000391)
Supplement: SUPPLEMENTARY MATERIAL [file lgt-22-219-s002.docx]

Supplemental table 1. Number of positive cells per matrix by simulation condition (8-11% and 17-23%).

| Simulation condition (%) | Range of results | Range for 95% of results | Median | Mode |
| --- | --- | --- | --- | --- |
| 8 | 0-19 | 3-13 | 7 | 7 |
| 9 | 1-21 | 3-14 | 8 | 8 |
| 10 | 1-20 | 4-15 | 9 | 9 |
| 11 | 1-25 | 5-16 | 10 | 9 |
| 17 | 4-28 | 9-22 | 15 | 15 |
| 18 | 4-30 | 9-24 | 16 | 16 |
| 19 | 4-32 | 10-25 | 17 | 17 and 18 |
| 20 | 6-33 | 11-26 | 18 | 19 |
| 21 | 6-35 | 12-27 | 19 | 19 |
| 22 | 6-35 | 12-28 | 20 | 20 |
| 23 | 7-37 | 13-29 | 21 | 20 |
