# Supplementary material for: Statistical Modeling for Quality Assurance of Human Papillomavirus DNA Batch Testing
Source: J Low Genit Tract Dis. 2018 May 4;22(3):219–24. doi: 10.1097/LGT.0000000000000391 (PMC6023602; doi:10.1097/LGT.0000000000000391)
Supplement: SUPPLEMENTARY MATERIAL [file lgt-22-219-s003.docx]

Supplemental table 2: Results for number of clusters per matrix by simulation condition (8-11% and 17-23%).

| Simulation condition (%) | Range of results | Range for 95% of results | Median | Mode |
| --- | --- | --- | --- | --- |
| 8 | 0-5 | 0-3 | 1 | 0 |
| 9 | 0-6 | 0-3 | 1 | 1 |
| 10 | 0-7 | 0-3 | 1 | 1 |
| 11 | 0-7 | 0-4 | 1 | 1 |
| 17 | 0-9 | 0-6 | 3 | 3 |
| 18 | 0-9 | 1-6 | 3 | 3 |
| 19 | 0-9 | 1-6 | 3 | 3 |
| 20 | 0-10 | 1-7 | 4 | 3 |
| 21 | 0-10 | 1-7 | 4 | 4 |
| 22 | 0-11 | 1-7 | 4 | 4 |
| 23 | 0-10 | 1-7 | 4 | 4 |
